# The Role of the Chloroplast in the Replication of Positive-Sense Single-Stranded Plant RNA Viruses

**DOI:** 10.3389/fpls.2018.01776

**Published:** 2018-11-27

**Authors:** Marta Budziszewska, Aleksandra Obrępalska-Stęplowska

**Affiliations:** Department of Entomology, Animal Pests and Biotechnology, Institute of Plant Protection – National Research Institute, Poznań, Poland

**Keywords:** replication, chloroplast, chloroplast membrane, viral replication complex (VRC), plant-virus interaction, viroids

## Abstract

Positive-sense single-stranded plant RNA viruses are obligate intracellular parasites that infect many agriculturally important crops. Most known plant RNA viruses are characterized by small genomes encoding a limited number of multifunctional viral proteins. Viral pathogens are considered to be absolutely dependent on their hosts, and viruses must recruit numerous host proteins and other factors for genomic RNA replication. Overall, the replication process depends on virus–plant protein-protein, RNA–protein and protein–lipid interactions. Recent publications provide strong evidence for the important role of chloroplasts in viral RNA synthesis. The chloroplast is considered to be a multifunctional organelle responsible for photosynthesis and for the generation of plant defense signaling molecules. High-throughput technologies (genomics and proteomics), and electron microscopy, including three-dimensional tomography, have revealed that several groups of plant RNA viruses utilize chloroplast membranes to assemble viral replication complexes (VRCs). Moreover, some chloroplast-related proteins reportedly interact with both viral proteins and their genomic RNAs and participate in trafficking these molecules to the chloroplast, where replication occurs. Here, we present the current knowledge on the important role of chloroplasts in the viral replication process.

## Introduction

Positive-sense, single-stranded [(+)ssRNA] viruses are the main agents of many serious diseases of agriculturally important crops. Despite their relatively small genomes, these viruses that encode only a few proteins can efficiently replicate in their host cells (Hyodo and Okuno, 2016). Viruses have evolved the ability to harness and reprogramme host cell metabolism, facilitating their propagation and the infection process and suppressing plant defense mechanisms (Nagy and Pogany, 2012; Wang and Li, 2012; de Castro et al., 2013). Indeed, viruses are able to hijack host factors, such as proteins, intracellular membranes and their modifiers, lipids, and metabolites, leading to the establishment of viral replication complexes (VRCs), sites where viruses assemble and mature (Nagy and Pogany, 2012). These viral factories may utilize organelles, such as the endoplasmic reticulum (ER), mitochondria, vacuoles, Golgi apparatus, peroxisomes, tonoplasts, and chloroplasts, as well as the plasma membrane (Sanfaçon, 2005; Nagy and Pogany, 2012; Xu and Nagy, 2014; Hyodo and Okuno, 2016). Xu and Nagy (2014) reviewed that members of virus genera, including tobamoviruses (tomato mosaic virus), potyviruses (tobacco etch virus, TEV; turnip mosaic virus, TuMV), and tombusviruses (tomato bushy stunt virus, TBSV), exhibit flexibility in the selection of subcellular compartments for viral factory biogenesis, suggesting that these viruses can utilize multiple organelles for replication, depending on the conditions or host species. Notably, results obtained mainly using plant viruses that are able to replicate in yeast, as exemplified by TBSV, showed that in the absence of peroxisomes, which are considered the primary site of replication, the ER provides an alternative subcellular membrane and that viruses are able to replicate on the ER surface. These findings highlight that membrane availability is a critical factor for virus replication (Jonczyk et al., 2007).

Despite their various locations, VRCs share common features such as comprising invaginated spherules/vesicles that are connected to the cytoplasm *via* a neck-like opening and single- and/or double-membrane systems (Grangeon et al., 2012; Jin et al., 2018b). Due to the development of advanced technologies in cellular, molecular, and structural biology, the identification of co-opted host factors and the biogenesis of viral factories are being widely studied, and chloroplasts have been found to be among the most important organelles for viral RNA synthesis. Accordingly, there is increasing interest in understanding the role of chloroplasts in virus replication.

The chloroplast is considered the most dynamic organelle in a plant cells. It is responsible for photosynthesis and plays an active role in antiviral defense involving the synthesis of phytohormones, including salicylic acid, and jasmonic acid, as well as the production of reactive oxygen species (ROS); chloroplasts are also crucial for interorganellar signaling (Bhattacharyya and Chakraborty, 2018). Zhao et al. (2016) reviewed a number of studies showing that chloroplasts are susceptible to damage during virus infection, leading to disease symptoms such as mosaics and chlorosis resulting from cytopathogenic effects in chloroplast structure or function. Additionally, chloroplast factors are known to participate in the movement of viral particles.

In this review, we describe chloroplast–virus interactions on the basis of recent evidence highlighting the direct function of this organelle in virus RNA synthesis. We also list the most useful molecular approaches for studying viral RNA replication in this context.

## Requirement of the Chloroplast Membrane for Plant ss(+)Rna Virus Replication

**FIGURE 1:**
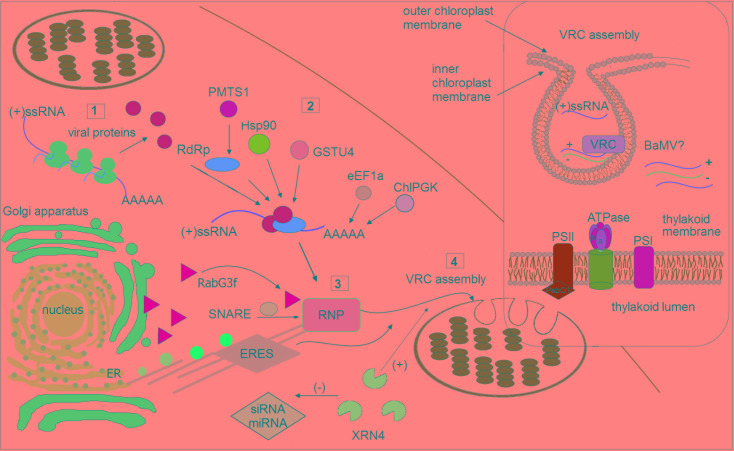
Schematic illustration of selected host factors affecting viral RNA replication in chloroplasts. Synthesis of viral genomic RNA [(+)ssRNA] requires viral proteins: RdRp (pink circle) and auxiliary factors (green circles) that are translated at the early stage of infection (1). Then, virus recruits host factors (2) such as chaperone (Hsp90), methyltransferase (e.g., PMTS1), chloroplast phosphoglycerate kinase (chlPGK), which participate in viral replication complexes (VRC) targeting (in potexvirus). The 3′UTR of viral RNAs (in potexviruses, e.g., BaMV) interacts with several cellular proteins, including elongation factor 1a (eEF1a), glutathione S-transferase (GSTU4), exonuclease (XRN4). Exonuclease negatively regulates the activity of silencing pathway (miRNA/siRNA). Subsequently, the hijacked host factors, viral genomic RNA and viral proteins are trafficked to chloroplast membranes (3). The ribonucleoprotein complex (RNP) is transported from endoplasmic reticulum (ER) exit sites ERES (in potyvirus) or via targeting proteins, such as Rabs or SNARE (in potexvirus) and actomyosin motility system. Chloroplast membranes are later reorganized to form VRC (4). The panel on the right depicts spherule where the viral RNA is synthesized. The host photosynthetic protein PsbO1 from photosystem II is recruited by potyvirus proteins playing a role in its replication (TVBMV, for details refer to the text). In thylakoid membranes, the β subunit of ATPase has been also involved in VRC assembly (potexvirus and tymovirus). Among viruses, viral RNA of BaMV was observed inside the chloroplast. The presented host factors participate in trafficking to the site of replication or affect (directly or indirectly) the synthesis of viral RNA, that is associated with chloroplast.

Although ss(+)RNA viruses display various genetic organization and expression strategies, they all replicate in a similar manner. All known plant ss(+)RNA viruses encode RNA-dependent RNA polymerase (RdRp) and ancillary replication factors. These viral proteins, which are produced in the early stage of infection, are involved in the assembly of VRCs and help to stabilize and facilitate optimal conditions for RNA synthesis. Viral and host proteins are selectively bound to the ss(+)RNA template and target subcellular membranes, which are later reorganized around the favorable microenvironment to form VRCs (Jin et al., 2018a). Within VRCs, negative-strand RNA intermediates are synthetized and then used as templates for the synthesis of new (+)RNA strands (Figure 1). The progeny RNAs are then released into the cytosol, where they might again be translated and/or replicated or encapsidated and transferred to adjacent cells. Indeed, the organellar compartmentalization of VRCs protects viral RNAs from being destroyed by host defense RNA degradation while simultaneously maximizing the concentration of viral and host factors required for replication (Nagy and Pogany, 2012). Previously published data reveal that chloroplasts serve as replication sites for several viruses, including hordeiviruses (barley stripe mosaic virus, BSMV) (Carroll, 1970; Torrance et al., 2006), potyviruses [maize draft mosaic virus (MDMV) (Mayhew and Ford, 1974), plum pox virus (Martin et al., 1995), TEV (Gadh and Hari, 1986)], TuMV (Kitajima and Costa, 1973), and tymoviruses (turnip yellow mosaic virus, TYMV) (Lafleche et al., 1972; Dreher, 2004). De Graaff et al. (1993) reported that VRCs of alfalfa mosaic virus (AMV), belonging to the *Alfamovirus* genus, are also associated with the chloroplast envelope. However, further studies performed by van der Heijden et al. (2001) and Ibrahim et al. (2012) using live cell imaging of *Arabidopsis thaliana* protoplasts showed that AMV replicates on tonoplast membranes.

The replication complex of the aforementioned virus families is associated with the outer chloroplast membrane, particularly with peripheral vesicles and cytoplasmic invaginations (CIs) on the surface. Among recently identified host factors participating in the viral replication process are several chloroplast and chloroplast-related proteins (Table 1). Virus-induced membrane proliferation and remodeling are very complex processes, and all eukaryotic (+)RNA viruses induce an interaction network between host and viral factors to form VRCs in subcellular membranes (Nagy and Pogany, 2012; Hyodo and Okuno, 2014). In addition to viral RNA and ancillary replication factors, viruses recruit multiple host RNA-modifying enzymes, translation factors, RNA helicases, including DEAD-Box RNA helicases (in plants and yeast), and methyltransferase. The VRCs of different plant virus families reportedly might contain the following components: chaperones (facilitating proper folding of RNA), viral RNA, and proteins, host endosomal sorting complex required for transport (ESCRT) that participates also in membrane deformation (Nagy and Pogany, 2012; Hyodo and Okuno, 2014; Geng et al., 2017), as well as other cellular proteins involved in lipid metabolism and transport (Barajas et al., 2014).

**Table 1:** List of host chloroplast and chloroplast-related factors and viral protein interactions involved in Synthesis of viral genomic RNA [(+)ssRNA] plant virus replication.

Host factors	Viral proteins/RNA	Viruses	Identified function in replication	Reference
NbRabG3f	–		VRC membrane trafficking	Huang et al., 2016
ChlPGK	RNA 3′UTR	potexvirus, BaMV	VRC membrane trafficking	Cheng et al., 2013
Hsp90	Viral polymerase RdRp/RNA3′UTR		VRC assembly	Huang et al., 2017
CA	–		initiation of RNA synthesis	Chen et al., 2017
GAPDH	RNA/satRNA 3′UTR		inhibition of the minus strand RNA synthesis (negative regulation)	Huang et al., 2017
PMTS1	Viral RdRp		an inhibitory effect on RdRp activity	Cheng et al., 2009
	VRC		targeting VRC to chloroplasts	
GSTU4	RNA 3′UTR		activation of minus-strand RNA synthesis	Chen et al., 2013
			a role in the antioxidation process	Huang et al., 2017
XRN4	VRC		enhancement of the accumulation of virus	Huang et al., 2017
Syp71	chloroplast- bound 6K2 complex	potyvirus, TuMV	assembly of VRC; mediation in the fusion of vesicles with chloroplast	Wei et al., 2013
Chloroplast membrane lipids	TGB2	pomovirus, PMTV	VRC membrane trafficking and assembly	Cowan et al., 2012
NbPsbO1	6K2	potyvirus, TVBMV, PVY	positive regulation of RNA synthesis	Geng et al., 2017
ATP- synthase β subunit	TGB1	potexvirus, AltMV	VRC assembly	Nam et al., 2012
ATP- synthase β subunit	–	tymovirus, ToBMV	VRC assembly	Megias et al., 2018
RNA polymerase beta-subunit	-		unknown	
eEF1a	RNA 3′UTR	potyvirus, TuMV	role unknown, the colocalization with 6K	Hyodo and Okuno, 2014
		potexvirus, BaMV	inhibition of (−)RNA synthesis	Huang et al., 2017

Recently published data indicate the role of the 140K/98K protein domains of TYMV, which possess two amphipathic helices (αA and αB) with a 41-residue internal sequence described as the chloroplast envelope-targeting domain (CTD) (Moriceau et al., 2017). Based on the results of structural analyses involving nuclear magnetic resonance (NMR) spectroscopy and molecular dynamics simulations, it was reported that amphipathic helices within viral proteins might serve as membrane-anchoring domains able to promote changes in lipid organization, thereby modulating membrane bilayer properties. However, in TYMV, the detailed delivery mechanisms remain to be elucidated (Moriceau et al., 2017). Nonetheless, Jin et al. (2018b) published data for 3D electron tomography (ET) characterization of the architecture of BSMV replication factories and revealed that chloroplast invaginations are composed of inner chloroplast membrane-derived packets containing variable numbers of outer membrane-derived spherules. Furthermore, studies by Torrance et al. (2006) and immunolocalization experiments by Zhang et al. (2017) show that BSMV genomic RNAs, replicative dsRNA intermediates, triple gene block 2 (TGB2) and viral γb proteins accumulate at chloroplasts. Importantly, the γa protein possesses RdRp activity and interacts with λa to induce the replication process. λa, which contains highly conserved methyltransferase and helicase domains, also plays a significant role in chloroplast membrane rearrangement. It has been shown that λa binds directly to γb and that this interaction is absolutely necessary for chloroplast targeting. Moreover, the γb protein is reported to enhance the helicase activity of λa (Zhang et al., 2017; Jin et al., 2018b).

In potyviruses, for example TEV and TuMV, the transport of viral proteins and genomic RNA to sites of replication occurs *via* vesicles from ER-exit sites (ERESs) to the outer chloroplast membrane (Wei and Wang, 2008; Wei et al., 2010). In addition, a model of the biogenesis and trafficking of viral 6K2 vesicles has been proposed based on confocal microscopy imaging (Wei et al., 2010). Potyvirus 6K2, an integral membrane protein, induces the formation of ER-derived vesicles that traffic predominantly from the ER to the Golgi apparatus and then target chloroplasts depending on the activities of the coat protein complexes COPI and COPII as well as the actomyosin motility system (Wei et al., 2010, 2013; Hyodo and Okuno, 2014; Zhao et al., 2016; Bhattacharyya and Chakraborty, 2018; Jin et al., 2018a; Figure 1). Fusion of virus-induced vesicles with chloroplast membranes and also chloroplast amalgamation rely directly on SNARE proteins (soluble NSF (*N*-ethylmaleimide – sensitive factor) attachment protein receptor) that are essential host factors for successful virus infection. For example, the cellular SNARE protein Syp71 directly mediates the fusion of vesicles with chloroplasts (Wei et al., 2013; Figure 1).

Cowan et al. (2012), performed lipid interaction assays using confocal laser scanning microscopy and biochemical analysis to determine that chloroplast membrane lipids associate with viral TGB2 of potato mop-top virus (PMTV), facilitating localization of viral genomic RNA to chloroplast membranes for replication. These findings confirmed that PMTV induces large CIs in chloroplasts, though the detailed ultrastructure has not yet been determined (Cowan et al., 2012). Moreover, fluorescence *in situ* hybridization studies of alternanthera mosaic virus (AltMV, a member of the genus *Potexvirus*) showed that viral RNA and TGB3, which possesses a chloroplast-targeting signal, primarily accumulate near the surface of chloroplast membranes (Lim et al., 2010). Further analyses proved that the TGB1 protein interacts with host chloroplast β-ATPase in *Nicotiana benthamiana* and *A. thaliana* (Nam et al., 2012). These data suggest that AltMV replication occurs at the outer chloroplast membrane. Additionally, interaction with the ATP-synthase β subunit was indicated by chloroplast proteome analyses of *N. benthamiana* plants infected with a member of the *Tymovirus* genus, tomato blistering mosaic virus (ToBMV), with VRCs adjacent to the chloroplast membrane (Megias et al., 2018). Moreover, in plants infected with ToBMV, increased expression of the chloroplast RNA polymerase β subunit was also observed during ToBMV infection. Considering that viruses encode RNA polymerases, the role of the increased gene expression of chloroplast polymerase during ToBMV infection remains unknown (Megias et al., 2018). Importantly, nuclear-encoded chloroplast RNA polymerases have a direct function in the replication of viroids of the family *Avsunviroidae* (Navarro et al., 1999, 2000; Daròs and Flores, 2002). These viroids are noncoding, circular RNAs with hammerhead ribozyme activity that accumulate and replicate in chloroplasts (Flores et al., 2005). Overall, the mechanism of viroid transport is still being studied. Gómez and Pallás (2010, 2012) suggest that avsunviroids are at first translocated to the nucleus and then employ a nucleus-chloroplast transport mechanism to enter chloroplasts for replication. Members of the *Avsunviroidae* replicate through a symmetric rolling circle mechanism, in which RNA circularization is catalyzed by chloroplastic tRNA ligase (Nohales et al., 2012; Cordero et al., 2018). Viroids hijack host factors for efficient replication, as do viruses, and such host factors as an aminomethylotransferase, chaperones, a dynamin-like guanosine triphosphatases (GTP)-binding protein, ribosomal protein L5, and elongation factor eEF1a, have been identified as binding to peach latent mosaic viroid (PLMVd) *in vitro* (Dubé et al., 2009); however, their importance for pathogen replication remains unclear. The results obtained through analyses of the eEF1a–PLMVd interaction suggest its role in the initiation of viroid replication, as occurs for several viruses, e.g., TYMV (Joshi et al., 1986) and BMV (Bastin and Hall, 1976). In addition, Daròs and Flores (2002) showed that RNA-binding proteins PARBP33 and PARBP35 (from avocado) bind to the RNA of avocado sunblotch viroid, the type member of the *Avsunviroidae*, and act as chaperones that presumably assist and stimulate self-cleavage.

## The Role of Host Proteins in Regulating the Viral Replication Process in Chloroplasts

Cheng et al. (2013) have reported interesting results concerning virus replication sites, showing that the minus-strand RNA of bamboo mosaic virus (BaMV, a potexvirus) is found not only in the organellar membrane but also throughout the chloroplast. Chloroplast protein phosphoglycerate kinase (ChlPGK) may also participate in targeting of VRCs to chloroplasts. Indeed, PGK interacts with the 3′ untranslated region (UTR) of the BaMV genomic RNA, and, together with the host heat shock proteins (Hsp90), translocates the viral ribonucleoprotein complex (RNP) across chloroplast membranes to the stroma and then stimulates VRC assembly in association with the thylakoid membranes (Cheng et al., 2013). Screening analyses of differentially expressed genes in plants during BaMV infection have indicated that NbRabG3f is up-regulated. Rabs comprise a group of small GTPases that regulate membrane trafficking, including vesicle budding, delivery, tethering, and fusion with target compartment membranes (Huang et al., 2016). BaMV may employ NbRabG3f to form vesicles derived from the Golgi membrane for intracellular trafficking to deliver factors to its replication site (Figure 1). GTPase activity and membrane-targeting ability are both crucial for virus replication (Huang et al., 2017).

The available evidence indicates that some host factors involved in viral replication in chloroplasts may enhance or inhibit this process. Analyses of the host factors assisting in BaMV replication have revealed at least three proteins, together with the abovementioned ChlPGK and Hsp90 may interact with the 3’ end of the viral genomic RNA. Among them are the eEF1a, glutathione S-transferase NbGSTU4 (from *N. benthamiana*), and exonuclease XRN4, which all play a crucial role in the early stage of viral replication (Cheng et al., 2013; Chen et al., 2017; Huang et al., 2017; Figure 1). For instance, XRN4 functions in reducing the activities of siRNA- and miRNA-mediated RNA silencing pathways in *Arabidopsis* (Souret et al., 2004). Importantly, NbXRN4 acts as an antiviral agent that attenuates accumulation of tobacco mosaic virus and tomato bushy mosaic virus. In contrast, Lee et al. (2016) indicated an enhancer role for NbXRN4 in BaMV replication, which is also exhibited in the presence of the universal silencing suppressor P19. Indeed, Huang et al. (2017) suggested that during viral RNP trafficking to chloroplasts, NbXRN4 may reduce the activities of siRNA-mediated silencing and, consequently, positively regulate BaMV RNA accumulation; this view, however, should be confirmed experimentally. It is worth noting that VRCs require antioxidant enzymes once the viral BaMV RNP complex is transported to the chloroplast, which is the major producer of (ROS) in plant cells. Oxidative stress may be attenuated *via* enzymatic activity by glutathione transferases (GSTs), and the 3′UTR-associated protein NbGSTU4 that in the presence of glutathione is able to neutralize oxidative stress inside chloroplasts, enabling efficient minus-strand RNA synthesis (Huang et al., 2017). It has also been documented that the initiation and elongation of viral BaMV RNA synthesis is positively regulated by nuclear-encoded plastidic carbonic anhydrase (CA), with the knockdown of this gene reducing accumulation of the BaMV coat protein (Chen et al., 2017). Furthermore, an increased level of CA was also observed by Obrepalska-Steplowska et al. (2015) during peanut stunt virus infection in *N. benthamiana*, which suggests that the observed differences in the expression levels of chloroplast-related genes might be dependent on the virus infection process.

Involvement of the oxygen-evolving complex in the regulation of virus replication has also been indicated. Geng et al. (2017) experimentally proved that down-regulation of NbPsbO1 expression suppressed the replication of tobacco vein banding mosaic virus (TVBMV) as well as PVY. Additionally, the 6K2 protein was recently reported to recruit 6K1 and hijack the chloroplast protein NbPsbO1, a component of the photosystem II oxygen-evolving complex that regulates potyvirus replication; consistently virus accumulation is significantly decreased in NbPsbO1-silenced plants.

The abovementioned examples show that viral pathogens hijack host factors for genome replication and production of new virions to ensure successful infection.

## Molecular Approaches to Identify and Characterize Host Factors Required for Plant ss(+)Rna Virus Replication

Identification of host factors involved in virus replication may be achieved *via* genome-wide screening approaches in combination with global proteomics analyses. A number of studies on viral–host protein interactions are based on a yeast two-hybrid approach, whereas host RNA-binding proteins may be identified using a viral RNA probe incubated with cellular extracts (with or without ultraviolet crosslinking), followed by mass spectrometry (Wang and Li, 2012). For identifying lipid factors, lipidomics assays demonstrating differences in lipid composition are very useful, and electron microscopy (EM) methods are extremely helpful for direct visualization of the particular steps of the life cycle of a given virus. Moreover, the development of three-dimensional EM (3D-EM) in combination with ET approaches provides unprecedented insights into how viruses remodel the intracellular architecture of the host cell. Indeed, 3D imaging techniques applied for viral replication factory reconstruction have been described for BSMV, TBSV, beet black scorch virus, melon necrotic spot virus (the *Tombusviridae* family), potyviruses TuMV and TEV, and *Bromoviridae,* as exemplified by brome mosaic virus (Jin et al., 2018a,b). All these innovative approaches together with molecular *in situ* mapping are powerful tools that provide essential architectural information regarding VRC biogenesis and the function of these structures (de Castro et al., 2016; Jin et al., 2018a).

## Conclusion

In summary, the abovementioned research studies broaden our knowledge of chloroplasts and their important direct and indirect roles in the replication of several plant virus species. In recent years, increasing numbers of chloroplast and chloroplast-related proteins have been shown to be involved in the formation of membranous replication factories and RNA synthesis. However, it is worth noting that in many cases the mechanism of interactions between viral and the identified plant factors as well as their influence on the regulation of replication remains unknown. Importantly, it should be noted that despite being replication sites, chloroplasts are primarily implicated in plant defense against viruses. Therefore, molecular insights into pathways exploited by viral pathogens and the implementation of innovative powerful imaging techniques to study plant–virus interactions is of great importance. The results obtained might contribute to the development of new and effective antiviral strategies against existing and emerging plant virus diseases.

## Author Contributions

MB and AO-S conceived the review. MB wrote a large part of the manuscript. AO-S critically reviewed and complemented the review. Both authors have made a substantial, direct and intellectual contributions to the work, and approved it for publication.

## Conflict of Interest Statement

The authors declare that the research was conducted in the absence of any commercial or financial relationships that could be construed as a potential conflict of interest.
